# Low miR-1273a expression predicts poor prognosis of colon cancer and facilitates tumor cell proliferation, migration, and invasion

**DOI:** 10.1590/1414-431X202010394

**Published:** 2021-01-08

**Authors:** Lei Sun, Xin Zhou, Qian Jiang, Yiping Zhuang, Dongzheng Li

**Affiliations:** 1 Invasive Technology Department, Jiangsu Cancer Hospital, Nanjing, Jiangsu, China; 2 Rectal Surgery Department, Jiangsu Cancer Hospital, Nanjing, Jiangsu, China; 3 General Surgery Department, Jiangsu Cancer Hospital, Jiangsu Institute of Cancer Research, Nanjing Medical University Affiliated Cancer Hospital, Nanjing, Jiangsu, China

**Keywords:** MicroRNA-1273a, Colon cancer, Prognosis, Proliferation, Migration, Invasion

## Abstract

MicroRNAs (miRNAs) have been indicated to be frequently dysregulated in various cancers and promising biomarkers for colon cancer. The present study aimed to assess the prognostic significance and biological function of miR-1273a in colon cancer. The expression levels of miR-1273a was estimated using quantitative real-time polymerase chain reaction. Kaplan-Meier survival curves and Cox regression analysis were used to evaluate the prognostic value of miR-1273a in patients of colon cancer. The effects of miR-1273a on cell proliferation, migration, and invasion were investigated by cell experiments. The expression of miR-1273a was downregulated in colon cancer tissues and tumor cell lines compared with the normal controls (all P<0.001). The aberrant expression of miR-1273a was associated with vascular invasion (P=0.005), differentiation (P=0.023), lymph node metastasis (P=0.021), and TNM stage (P=0.004). The patients with low miR-1273a expression had low overall survival compared with the patients with high miR-1273a expression (log-rank P=0.002). miR-1273a was detected to be an independent prognostic biomarker for patients. Furthermore, the results of cell experiments revealed that miR-1273a downregulation promoted, while miR-1273a upregulation suppressed the cell proliferation, migration, and invasion. In conclusion, all data indicated that a downregulated expression of miR-1273a predicted poor prognosis for colon cancer and enhanced tumor cell proliferation, migration, and invasion. Thus, we suggest that methods to promote miR-1273a expression may serve as novel therapeutic strategies in colon cancer.

## Introduction

Globally, colon cancer, which is one of the leading causes of cancer deaths, is the second most common cancer in women and the third in men (1,2). It has been reported that colon cancer is one of the most common malignant tumors in the digestive system, and the morbidity and mortality rates are high (3). A large number of studies have shown that the occurrence of colorectal cancer was associated with dietary habits, induced inflammation, and other factors such as genetic mutations (4–6). In developing countries, about a quarter of colon cancer patients are at an advanced stage of development and have lost the chance for radical surgery (7). Thus, despite the fact that colon cancer diagnosis and treatment have developed rapidly in recent years, colon cancer is still an important clinical challenge worldwide because of its high prevalence and poor prognosis (8). Therefore, searching for prognostic biomarkers and exploring effective treatment methods for human colon cancer are needed urgently.

MicroRNAs (miRNAs) are a group of endogenous, small (about 22 nucleotides) non-coding RNAs that negatively regulate gene expression (9). They inhibit gene expression by binding to the 3′-untranslated region (3′-UTR) of their target mRNAs (10). An increasing number of studies have reported that miRNAs are mainly involved in the development and progression of a wide variety of cancers (11). miRNAs are involved in almost all biological processes, and many of them have been identified to function as carcinogenic miRNAs or suppressive miRNAs (12). Due to the frequent dysregulation in cancers, miRNAs serve as attractive targets for prognostication and therapeutic applications (13). In recent years, there have been many studies that have explored the relationship between miRNAs and colon cancer (14–16), but there is no study that has explored the relationship between microRNA-1273a (miR-1273a) and colon cancer. However, a study has reported that circRNA PIP5K1A can promote the development of colon cancer by inhibiting miR-1273a, suggesting that miR-1273a may mediate the biological function of circRNA PIP5K1A in colon cancer (17).

In this study, we explored the expression of miR-1273a in colon cancer tissues and cells, and assessed its prognostic value for cancer patients and the biological function of miR-1273a in the cells. The result of this study may provide evidence for miR-1273a to serve as a potential biomarker and therapeutic target in the treatment of colon cancer.

## Material and Methods

### Patients and tissue collection

A total of 124 patients who were pathologically diagnosed with colon cancer and underwent resection surgery in Jiangsu Cancer Hospital between 2011 and 2014 were recruited for this study. None of the patients had previously received any anti-tumor therapy. The experimental procedures of this study were approved by the Ethics Committee of Jiangsu Cancer Hospital. The colon cancer tissues and adjacent normal tissues were collected from the patients during surgery, and were immediately frozen in liquid nitrogen for further use. All the enrolled patients had complete electronic medical record data, and their clinicopathological characteristics are summarized in Table 1. Informed consent was signed by each patient. A 5-year follow-up survey was arranged for each patient after the surgery, and their survival information was recorded.

**Table 1:** Association of miR-1273a expression level with clinical characteristics of colon cancer patients.

Features	(n=124)	miR-1273a expression		P values
		Low (n=66)	High (n=58)	
Age (years)				0.735
≤60	49	27	22	
>60	75	39	36	
Gender				0.827
Female	44	24	20	
Male	80	42	38	
Vascular invasion				0.005
Negative	78	34	44	
Positive	46	32	14	
Differentiation				0.023
High/moderate	70	31	39	
Poor	54	35	19	
Lymph node metastasis				0.021
Negative	72	32	40	
Positive	52	34	18	
TNM stage				0.004
I-II	64	26	38	
III-IV	60	40	20	

Chi-squared test.

### Cell culture and transfection

Four colon cancer cell lines (LoVo, HCT116, SW480, and SW620) and a normal colonic mucosa cell line (NCM460) were purchased from American Type Culture Collection (ATCC, USA). The cells were cultured using Dulbecco's modified Eagle's medium (DMEM) supplemented with 10% fetal bovine serum (FBS; Gibco, USA), and maintained in a 5% CO_2_ atmosphere at 37°C. MiR-1273a mimic and inhibitor and corresponding negative controls were purchased from GenePharma (China). The cells were seeded in 6-well plates with 5×10^5^ cells/well, transfected with the miR-1273a mimic, mimic negative control (NC), miR-1273a inhibitor, or inhibitor NC using Lipofectamine 2000 transfection reagent (Invitrogen, USA) according to the manufacturer's protocols. Cells were collected after transfection for 24 h and used for following analyses.

### RNA extraction and quantitative real-time PCR (qRT-PCR</emph>*)*

Total RNAs of cells and tissues were extracted using TRIzol reagent (Invitrogen) according to the manufacturer's protocols. The concentration and quality of RNAs were assessed with a NanoDrop 2000 (Thermo Scientific, USA). Synthesis of single-stranded cDNA was carried out using PrimeScript RT Reagent Kit (Takara, Japan).

The expression levels of miR-1273a were examined using qRT-PCR, which was performed by SYBR Green I Master Mix kit (Invitrogen) using a 7300 real-time PCR system (Applied Biosystems, USA). U6 was used as the internal control and the 2^-ΔΔCt^ method was used to calculate the relative quantification.

### Cell proliferation assay

After transfection, the stably transfected cells were seeded in 96-well plates with a cell concentration of 1000 cells/well. MTT (20 µL, 5 mg/mL; Beijing Solarbio Science & Technology Co., Ltd., China) was added to each well after 0, 24, 48, and 72 h incubation. After 4 h further incubation, the medium was removed and 100 µL dimethyl sulfoxide was added to each well to dissolve the formazan. The proliferation of surviving cells was assessed by measuring the absorbance at 570 nm.

### Cell migration and invasion assay

Colon cancer cell migration and invasion were assessed using the cell Transwell system (Corning, USA), which was performed using Transwell chambers and polycarbonate membranes with an 8-µm pore size. The membranes coated with Matrigel (Corning) were used for the invasion analysis, while the uncoated membranes were used for the migration analysis. The upper chambers with serum-free medium were seeded with 5×10^4^ transfected cells, and the bottom chambers were filled with medium containing 10% FBS. After incubation for 24 h, the cells remaining on the upper membrane surface were removed with cotton swabs, whereas cells that migrated or invaded onto the bottom chambers were fixed with 100% methanol and stained with crystal violet. Then, the cells in the bottom were counted using an inverted microscope.

### Statistical analysis

All experiments were repeated at least three times and the data are reported as means±SD. All statistical analyses were performed using SPSS 21.0 software (IBM, USA) and GraphPad Prism 7.0 software (USA). The data of two groups or multiple groups were compared using Student's *t*-test or ANOVA followed by Tukey's test. The relationship between miR-1273a expression and clinicopathological data was analyzed using chi-squared test. Survival analysis was performed using Kaplan-Meier methods and log-rank test. Prognostic value of miR-1273a was confirmed by Cox regression analysis. Differences with P<0.05 were considered statistically significant.

## Results

### Downregulation of miR-1273a in colon cancer tissues and cell lines

**Figure 1:**
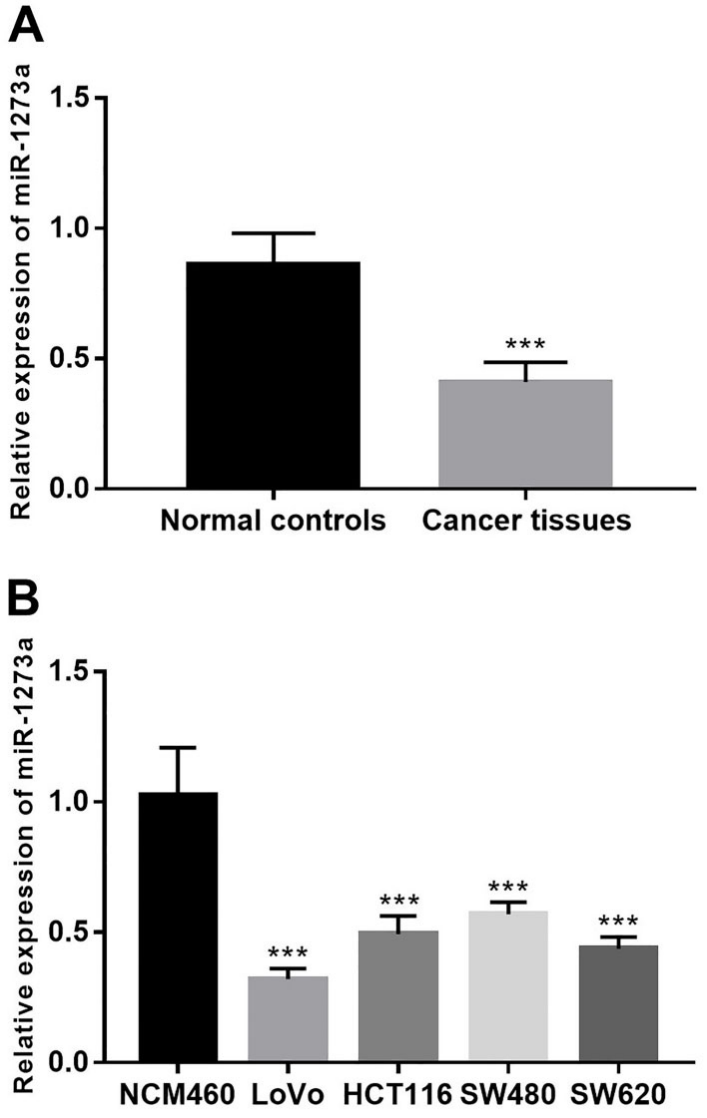
Expression of miR-1273a in colon cancer tissues and cell lines. **A**, The expression level of miR-1273a was downregulated in colon cancer tissues compared with the adjacent normal controls. **B**, The expression level of miR-1273a in colon cancer cell lines was lower than in the normal controls. Data are reported as means±SD. ***P<0.001 (Student's *t*-test or ANOVA followed by Tukey's test). NCM460: normal colonic mucosa cell line.

The expression of miR-1273a was significantly downregulated in colon cancer tissues compared with the adjacent normal tissues (P<0.001). Similarly, the reduced expression of miR-1273a was also observed in the four colon cancer cell lines compared with the normal cells (all P<0.001) (Figure 1).

### Association of miR-1273a with clinicopathological characteristics of colon cancer patients

Due to the dysregulated expression of miR-1273a investigated in colon cancer, we suspected that it might be involved in colon cancer development. Thus, this study investigated the relationship between miR-1273a and the clinicopathological data of cancer patients to examine the role of miR-1273a in colon cancer development. As shown in Table 1, the patients with colon cancer were divided into two groups (low and high miR-1273a expression) according to the mean value of miR-1273a expression. Notably, the expression of miR-1273a was markedly associated with vascular invasion (P=0.005), differentiation (P=0.023), lymph node metastasis (P=0.021), and TNM stage (P=0.004). However, there was no statistically significant relationship between miR-1273a expression and age or gender (all P>0.05).

### Clinical significance of miR-1273a in the prognosis of colon cancer

**Figure 2:**
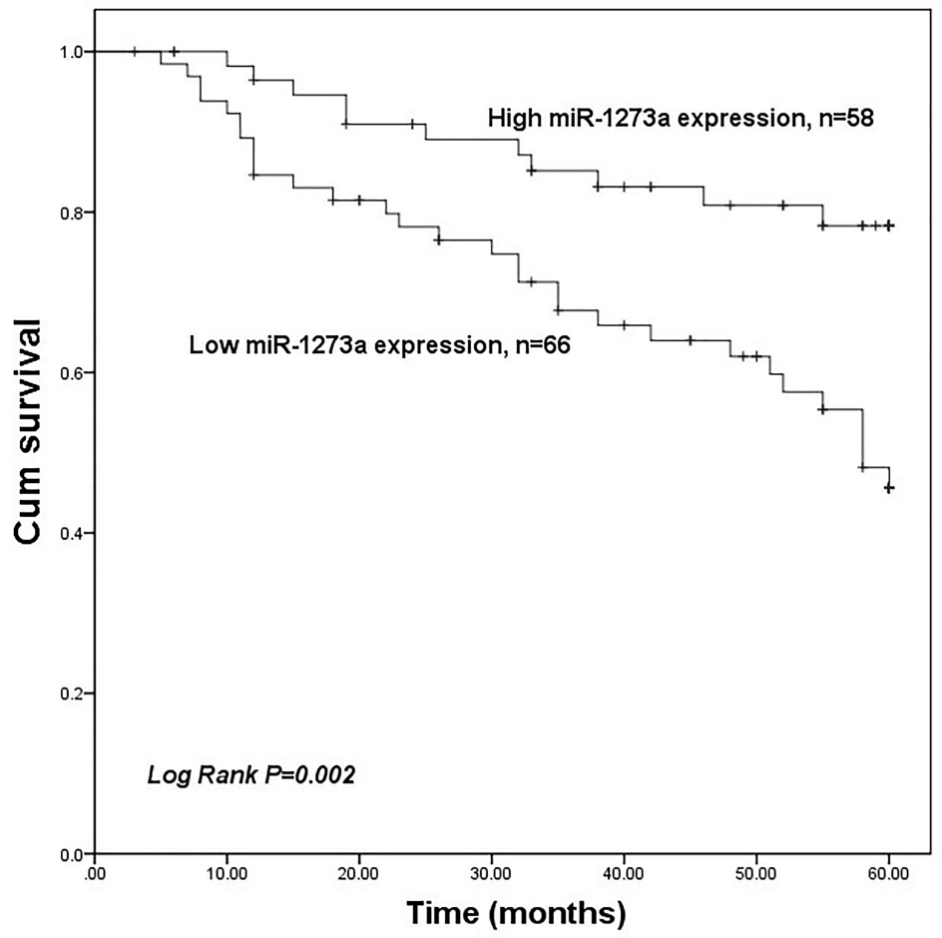
Kaplan-Meier survival curves for the patients with colon cancer. Overall survival in the patients with low miR-1273a expression was poor compared with the patients with high miR-1273a expression (log-rank P=0.002).

The overall survival was lower in patients with low miR-1273a expression compared with the patients with high miR-1273a expression (log-rank P=0.002) (Figure 2). Furthermore, the factors miR-1273a (HR=2.669, 95%CI=1.253-5.685, P=0.011), vascular invasion (HR=2.277, 95%CI=1.020-5.083, P=0.045), and TNM stage (HR=0.470, 95%CI=0.2270.975, P=0.043) were three independent prognostic factors for colon cancer patients (Table 2).

**Table 2:** Multivariate Cox regression analysis for miR-1273a in colon cancer patients.

Variables	Multivariate analysis		
	HR	95%CI	P value
miR-1273a	2.669	1.253-5.685	0.011
Age (years)	0.944	0.499-1.784	0.859
Gender	0.990	0.521-1.882	0.976
Vascular invasion	2.277	1.020-5.083	0.045
Differentiation	1.411	0.752-2.649	0.283
Lymph node metastasis	0.689	0.356-1.333	0.268
TNM stage	0.470	0.227-0.975	0.043

HR: hazard ratio.

### Effect of miR-1273a on proliferation of colon cancer cells

**Figure 3:**
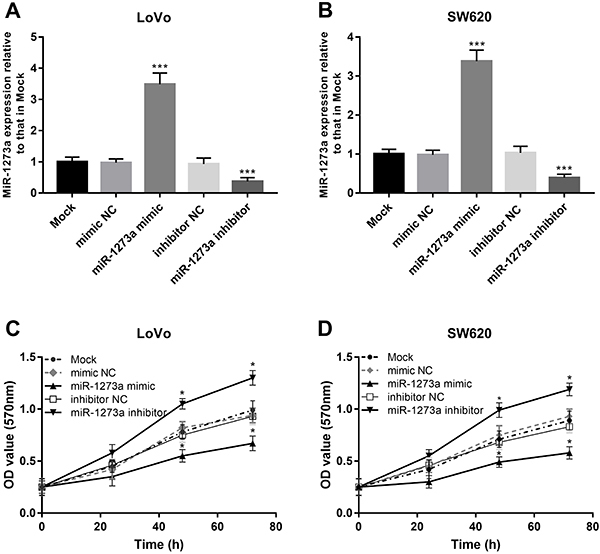
The expression of miR-1273a in (**A**) LoVo and (**B**) SW620 cell lines was significantly promoted by miR-1273a mimic, while it was significantly repressed by miR-1273a inhibitor compared with the untreated cells. The proliferation of both (**C**) LoVo and (**D**) SW620 cells was significantly promoted by miR-1273a downregulation, whereas it was significantly suppressed by miR-1273a upregulation. Data are reported as means±SD. *P<0.05, ***P<0.001 (ANOVA followed by Tukey's test). NC: negative control.

By qRT-PCR, we observed that the miR-1273a expression in LoVo and SW620 cell lines was increased in the cells transfected with miR-1273a mimic, while it was decreased in the cells transfected with miR-1273a inhibitor (all P<0.001, Figure 3A and B). Subsequently, the results of MTT indicated that miR-1273a overexpression inhibited, while the miR-1273a downregulation promoted the cell proliferation in both cell lines (all P<0.05, Figure 3C and D).

### Effect of miR-1273a on cell migration and invasion of colon cancer cells

**Figure 4:**
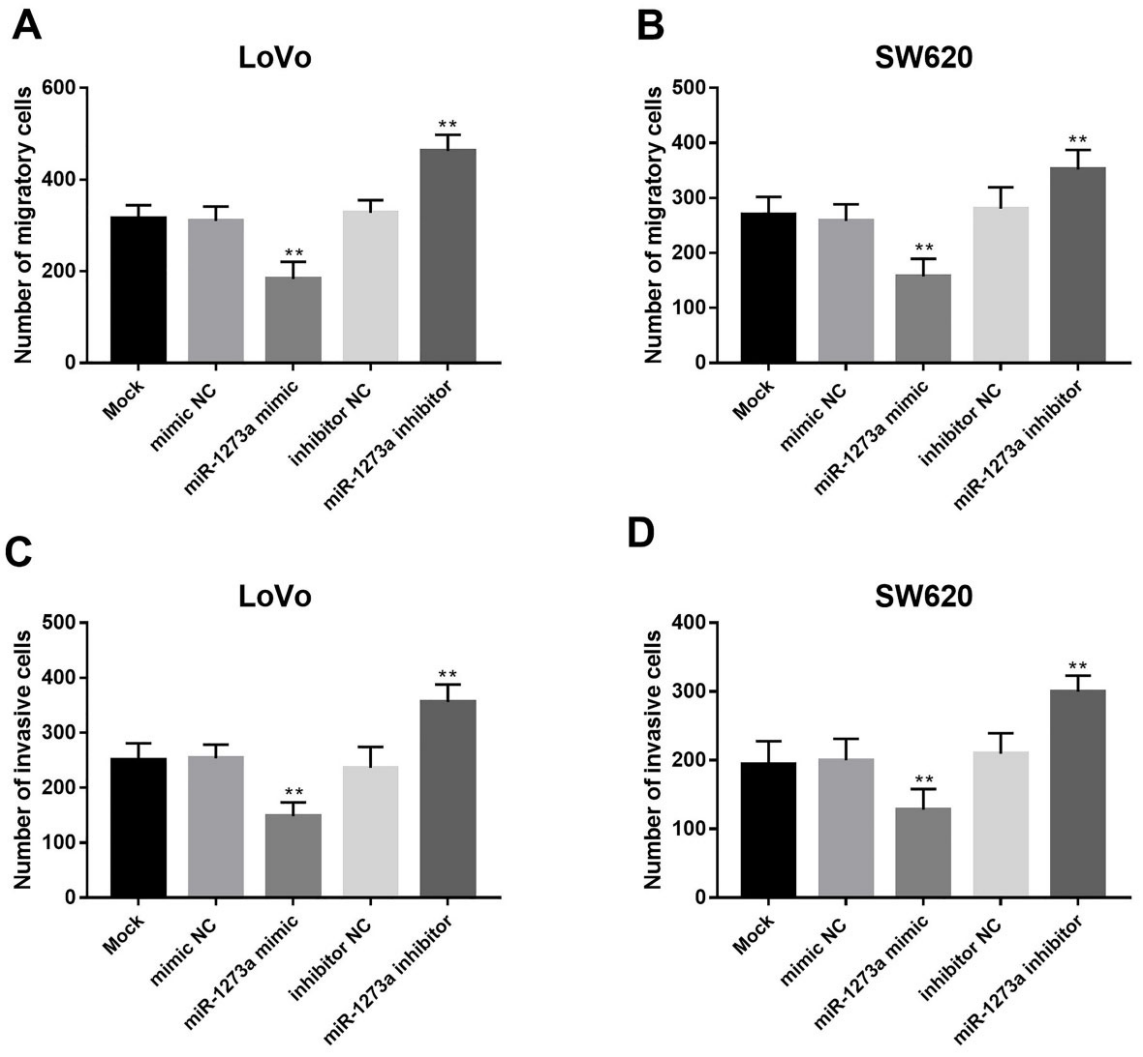
**A** and **B**, The upregulation of miR-1273a suppressed, whereas the downregulation of miR-1273a promoted, the migration of colon cancer cells. **C** and **D**, Similarly, the invasion of colon cancer cells was suppressed by miR-1273a upregulation, whereas it was promoted by miR-1273a downregulation. Data are reported as means±SD. **P<0.01 (ANOVA followed by Tukey's test).

As presented in Figure 4A and B, cell migration of both LoVo and SW620 cells was suppressed by miR-1273a upregulation, whereas it was promoted by miR-1273a downregulation (all P<0.01). Similarly, miR-1273a upregulation inhibited, whereas miR-1273a downregulation promoted, the invasion of both LoVo and SW620 cells (all P<0.01, Figure 4C and D).

## Discussion

miRNAs are small, noncoding RNAs that negatively regulate specific target genes expression (18). Growing evidence highlights their key roles played in various biological processes of human cancers (19), sometimes by enhancing cancer growth and other times by inhibiting tumorigenesis (20). For example, Mu et al. found that miR-31 expression is increased and promotes the proliferation and invasion of tumor cells (21). Fan et al. (22) found that miR-125a overexpression predicts poor prognosis and plays an anticancer role in cervical cancer. A study by Wu et al. (23) reported that miR-4458 inhibits tumor cells growth, migration, and invasiveness in breast cancer. These studies indicate that functional miRNAs can be used as a novel target for tumor targeted therapy.

Some miRNAs have been studied in colon cancer. For example, miR-378 has been reported to play an anticancer role in colon cancer progression, which could suppress the development of cancer by directly inhibiting target gene SDAD1 (24). MiR-590-3p promoted colon cancer progression via WIF1 and DKK1, and it might be a promising biomarker for colon cancer treatment (25). The aberrant expression of miR-122 has been indicated to be involved in cancer cells proliferation and invasion by inhibiting the expression of fructose-bisphosphate A aldolase (ALDOA) (26). Therefore, we predicted that miRNAs dysregulation played a key role in colon cancer.

In our study, we found that the expression of miR-1273a was downregulated in the colon cancer tissues and tumor cell lines compared with the adjacent normal cells. Additionally, miR-1273a expression was significantly associated with vascular invasion, differentiation, lymph node metastasis, and TNM stage. Therefore, we predicted that miR-1273a might be involved in the progression of colon cancer. Zhang et al. (17) found that miR-1273a overexpression abolishes the oncogenic function of cicrPIP5K1A in colon cancer.

Due to the dysregulation of miR-1273a in colon cancer, its clinical significance in the prognosis of colon cancer has also been studied. miRNAs have been found to be of high diagnostic and prognostic value in different types of human cancers (27–29). Some miRNAs have been found in colon cancer as biomarkers. For example, Li et al. (30) found that miR-195 is significantly decreased in cancer tissues and is a potential diagnostic marker for colon cancer. miR-370 was found to be decreased significantly in cancer tissues and may be a novel biomarker of colon cancer (31). Furthermore, we have found that miR-1273a expression was significantly decreased in colon cancer tissues. Thus, we hypothesized that miR-1273a might be a marker of colon cancer. Therefore, the prognostic value of miR-1273a was evaluated based on 5-year survival information of colon cancer patients. We found that the patients with low expression of miR-1273a had lower overall survival. Moreover, multivariate Cox analysis suggested that miR-1273a was an independent prognostic marker in colon cancer patients. Carcinoembryonic antigen (CEA) and carbohydrate antigen19-9 (CA199) as classic markers of colon cancer are directly related to the diagnosis and prognosis of colon cancer (32). A study has reported that CEA is a strong prognostic factor, and a high level of CEA indicates poor prognosis in stage II colon cancer (33). Zhou et al. (34) found that high levels of preoperative serum CA199 implies a poor prognosis for stage III patients with colon cancer. In this study, we demonstrated that low miR-1273a indicated poor prognosis of colon cancer. miRNAs are easy to detect, and we believe that the prognostic value of the classic protein markers may be improved significantly with the help of miRNAs in future clinical practices.

In order to further understand the biological function of miR-1273a in the progression of colon cancer, cell experiments were performed using miR-1273a mimics and inhibitors. After transfection, the expression of miR-1273a was upregulated by miR-1273a mimic, whereas expression was downregulated by miR-1273a inhibitor. Subsequently, the effects of miR-1273a on the biological functions of colon cancer cells were studied. The results indicated that the upregulation of miR-1273a inhibited, while the downregulation of miR-1273a promoted, the proliferation, migration, and invasion of tumor cells. The results of cell experiments suggested that miR-1273a had an anticancer effect in the progression of colon cancer.

Although we provided evidence that miR-1273a inhibited colon cancer progression, the underlying mechanisms remained unclear. Our study did not carry out *in vivo* experiments, which was a limitation. Therefore, it is necessary to further study the role of miR-1273a in colon cancer *in vivo* and explore its exact mechanism.

## Conclusion

In summary, the results of this study indicated that miR-1273a expression was decreased in colon cancer cells and could be used as a candidate biomarker for colon cancer prognosis. Overexpression of miR-1273a can inhibit the proliferation, migration, and invasion of colon cancer cells, suggesting it may be used as a therapeutic target for colon cancer.
